# Supplementary material for: Association between oral microbiome and seven types of cancers in East Asian population: a two-sample Mendelian randomization analysis
Source: Front Mol Biosci. 2023 Nov 21;10:1327893. doi: 10.3389/fmolb.2023.1327893 (PMC10702768; doi:10.3389/fmolb.2023.1327893)
Supplement: Supplementary file 1 [file DataSheet1.ZIP › Supplementary Materials/MR plots of saliva/saliva═╝/Breast cancer/pheno.208_to_breast cancer_forest.pdf]

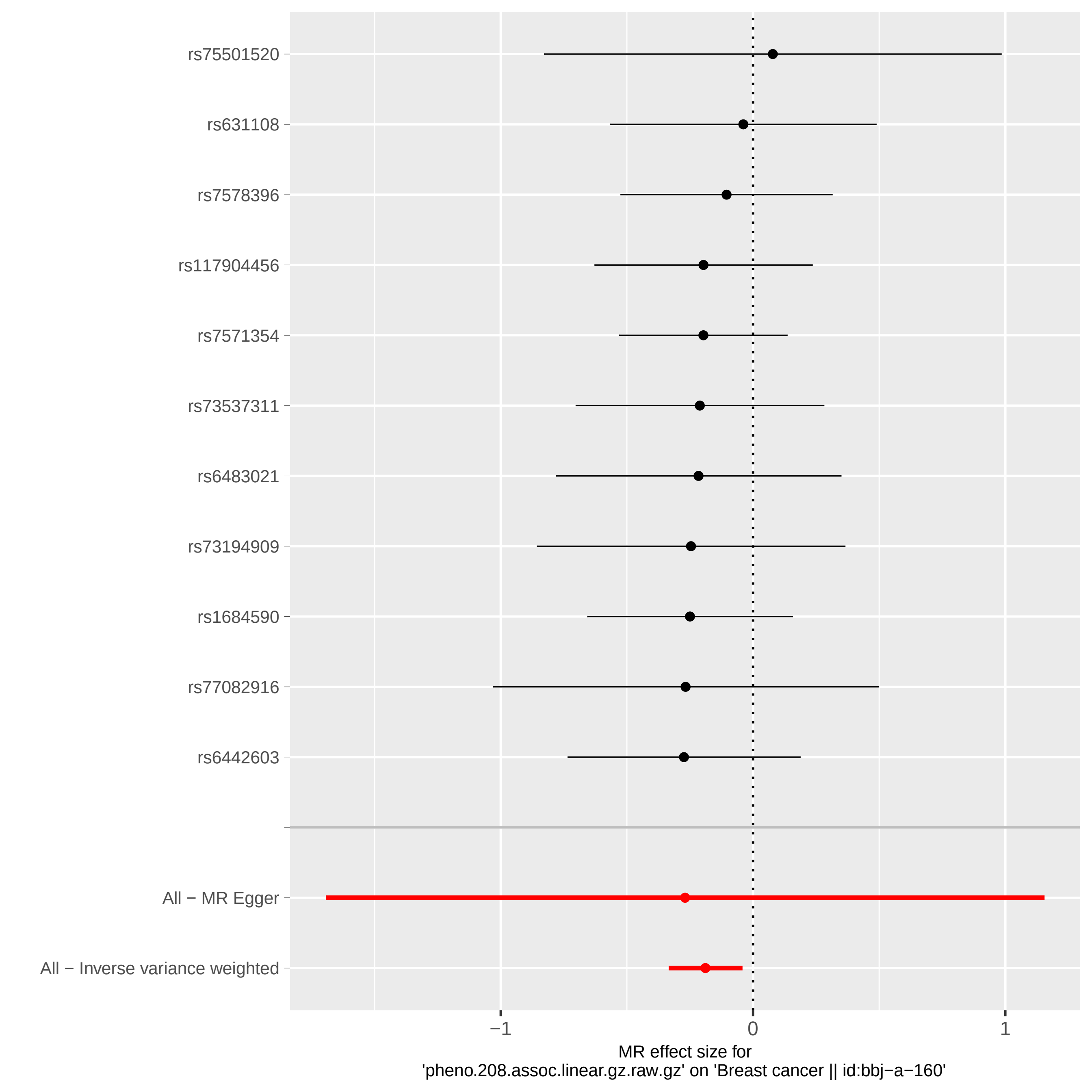
rs75501520
rs631108
rs7578396
rs117904456
rs7571354
rs73537311
rs6483021
rs73194909
rs1684590
rs77082916
rs6442603
All − MR Egger
All − Inverse variance weighted
−1
0
1
MR effect size for
'pheno.208.assoc.linear.gz.raw.gz' on 'Breast cancer || id:bbj−a−160'
